# Natural products as starting points for future anti-malarial therapies: going back to our roots?

**DOI:** 10.1186/1475-2875-10-S1-S3

**Published:** 2011-03-15

**Authors:** Timothy NC Wells

**Affiliations:** 1 Medicines for Malaria Venture, 20 rte de Pré-Bois, CH-1215 Geneva, Switzerland

## Abstract

**Background:**

The discovery and development of new anti-malarials are at a crossroads. Fixed dose artemisinin combination therapy is now being used to treat a hundred million children each year, with a cost as low as 30 cents per child, with cure rates of over 95%. However, as with all anti-infective strategies, this triumph brings with it the seeds of its own downfall, the emergence of resistance. It takes ten years to develop a new medicine. New classes of medicines to combat malaria, as a result of infection by *Plasmodium falciparum* and *Plasmodium vivax* are urgently needed.

**Results:**

Natural product scaffolds have been the basis of the majority of current anti-malarial medicines. Molecules such as quinine, lapachol and artemisinin were originally isolated from herbal medicinal products. After improvement with medicinal chemistry and formulation technologies, and combination with other active ingredients, they now make up the current armamentarium of medicines. In recent years advances in screening technologies have allowed testing of millions of compounds from pharmaceutical diversity for anti-malarial activity in cellular assays. These initiatives have resulted in thousands of new sub-micromolar active compounds – starting points for new drug discovery programmes. Against this backdrop, the paucity of potent natural products identified has been disappointing. Now is a good time to reflect on the current approach to screening herbal medicinal products and suggest revisions. Nearly sixty years ago, the Chinese doctor Chen Guofu, suggested natural products should be approached by *dao-xing-ni-shi* or ‘acting in the reversed order’, starting with observational clinical studies. Natural products based on herbal remedies are in use in the community, and have the potential unique advantage that clinical observational data exist, or can be generated. The first step should be the confirmation and definition of the clinical activity of herbal medicinal products already used by the community. This first step forms a solid basis of observations, before moving to *in vivo* pharmacological characterization and ultimately identifying the active ingredient. A large part of the population uses herbal medicinal products despite limited numbers of well-controlled clinical studies. Increased awareness by the regulators and public health bodies of the need for safety information on herbal medicinal products also lends support to obtaining more clinical data on such products.

**Conclusions:**

The relative paucity of new herbal medicinal product scaffolds active against malaria results discovered in recent years suggest it is time to re-evaluate the ‘smash and grab’ approach of randomly testing purified natural products and replace it with a patient-data led approach. This will require a change of perspective form many in the field. It will require an investment in standardisation in several areas, including: the ethnopharmacology and design and reporting of clinical observation studies, systems for characterizing anti-malarial activity of patient plasma samples *ex vivo* followed by chemical and pharmacological characterisation of extracts from promising sources. Such work falls outside of the core mandate of the product development partnerships, such as MMV, and so will require additional support. This call is timely, given the strong interest from researchers in disease endemic countries to support the research arm of a malaria eradication agenda. Para-national institutions such as the African Network for Drugs and Diagnostics Innovation (ANDi) will play a major role in facilitating the development of their natural products patrimony and possibly clinical best practice to bring forward new therapeutics. As in the past, with quinine, lapinone and artemisinin, once the activity of herbal medicinal products in humans is characterised, it can be used to identify new molecular scaffolds which will form the basis of the next generation of anti-malarial therapies.

## The need for new classes of medicines

Malaria remains one of the most significant health issues that we face today, with 250 million cases, and over 800,000 deaths annually. This impact is multiplied by the fact that 85% of the cases are in children under five years old, and the prevalence is high amongst expectant mothers. The current gold standard medicine is the fixed dose artemisinin combination therapy: consisting of chemical derivatives of the Chinese natural product artemisinin, and a longer acting partner (an arylamino alcohol or 4-amino quinoline), which ultimately traces its ancestry back to the natural product quinine. These medicines are extraordinary effective, curing more 98% of patients (measured 28 days after treatment, to make exclude patients who recrudesce). They are also relatively safe, often with no serious adverse events seen in phase III trials of several thousand patients. They are also relatively cheap: the cost of a cure for the smallest of children can be as low as $0.33 when purchased by the public sector in disease endemic countries. However, in the long term there will always be a need for new therapies – no matter how carefully the current medicines are used then there is the constant threat of resistance. The first signs of resistance to artemisinins are emerging, with patients taking longer to clear their fever and parasite in some parts of Cambodia [1]. This is an early warning sign that new classes of anti-malarials are needed, and are an urgent priority. It takes at least ten years to move a molecule from late in the discovery phase to completion of clinical trials, and there is a relative dearth of compounds with new mechanisms of action in clinical development at this time. Natural products and their derivatives have been a powerful part of the fight against malaria in the past – however, success requires that the lessons of the past are not disregarded.

## A revolution in screening: micromolar activity on its own is not enough

In the search for new medicines to combat malaria there have been two major technological steps forward over the last ten years. First, the parasite genomes have been sequenced, allowing, a systematic analysis of all the essential and ‘druggable’ genes. This has allowed assays to be set up for screening with the hope of finding new starting points for chemical programmes [2]. Second, advances in image processing and automation technology allow that assays for live parasites inside human host cells to be run in 384 or 1,536 well formats [3,4], making screening a hundred times faster and a hundred times cheaper than ten years ago. These technological advances have meant that the malaria community can now screen millions of compounds from the pharmaceutical industry, both in-house and also as collaborations with academic centres [5]. The results for malaria have been surprisingly good, with hit rates (compounds with confirmed IC50 values of less than 1 μM) of 0.5%. The frequency at which these cell-based screening hits occur is an important factor in analysing the best way to discover new medicines. For example, the hit rates in assays using live parasites in erythrocytes are higher those often found screening many classes of molecular targets. The live parasite approach is also one step ahead of target based approaches, in that the hits are already known to kill parasites, so have addressed cellular permeability question. They will also have been counter-screened against a human cell line, to show lack of toxicity [6]. The next important question for new hits is to know whether they will be active against existing drug-resistant strains of malaria, and this can be integrated into the cell biology at the outset. Where such clinical isolates do not exist, early information on the way resistance could occur to these new compounds can be rapidly obtained by *in vitro* generation of resistance, and sequencing. Compounds identified in this way can be successfully optimized without knowing the precise molecular target. Target identification can be performed in parallel to the lead optimisation process. Indeed, the molecules may have multiple molecular targets, and this would be an advantage in combating the emergence of resistance [7]. Cell-based screening has been a success. But the most important message is that now there are 25,000 distinct pure compounds with potencies against the parasite of less than one micromolar *in vitro* covering over a thousand different scaffolds, many of these in the public domain. These have been rapidly advanced to clinical candidates.

This resets the way that the relative success of herbal medicinal products approaches to anti-malarial drug discovery are measured. They have not fared nearly so well recently: only three hundred new anti-malarial compounds have been isolated from plants used in traditional medicine over the period 2005 to 2008 [8]. However, the reality is even more problematic, since these authors used a cut-off of 11 micromolar in their cellular assays. If the same cut-off used in pharmaceutical screening is applied, only 20 new structures pass, a much lower yield than from screening pharmaceutical diversity. Unfortunately, much of the subsequent natural products evaluation work in the literature is still focused on these low potency molecules; arguably an opportunity cost. Setting a more stringent cut-off makes sense from a number of other perspectives. First, clinical reality: few molecules are present in plasma with free concentrations above 1 μM for significant lengths of time in human. Second, as the potency cut-off is weakened, the chances of random interactions with the cell increase, opening up the possibility of toxicity [9]. The natural products will tend to have a low ligand efficiency, low potency and large number of heavy atoms [10], which makes them less attractive as starting points, (unless it is already known they are active in human). There are several purified natural products, which were identified as active molecules in the hits obtained by screening diversity from the pharmaceutical diversity collections. But even these have not yielded many useful new starting points [11], often only finding antibiotics with known anti-malarial activities, such as the macrolide family. In brief, the new-found ability to screen pharmaceutical diversity against parasites in malaria-infected cells suggests a reassessment of the standards for success in natural products based screening. Either a natural product hit must be dramatically more potent; (many of the oncology based natural products hits are single digit nanomolar), or it must have some other clearly definable advantage. One such major advantage would be knowing clearly from the start that the preparation worked in humans. That it has a defined clinical activity which could be reproducibly observed in malaria patients, and without obvious acute toxicology. This is most often overlooked.

## The historical importance of natural products

The history of anti-malarial chemotherapy is intimately linked with the history of herbal medicinal products. Quinine, the original natural product used in anti-malarial chemotherapy was identified from cinchona tree bark, and purified in 1820. The attempt to synthesize quinine led to the development of methylene blue and the dye industry. From this came the classical 4-aminoquinolines and amino-alcohols, such as chloroquine, amodiaquine and mefloquine, which have been the mainstay of malarial treatment over the last century. The natural product naphthoquinone lapichol also was identified as the active ingredient in tree bark used to treat malaria. This discovery helped direct the selection of lapinone, which in turn provided the foundation for the discovery of atovaquone, a component of Malarone®, still a mainstay of malaria prophylaxis for travellers [12]. Artemisinin was first isolated from the leaves of the sweet wormwood – *Artemisia annua* in 1971. The highly unusual endoperoxide group has formed the basis for a range of fully synthetic, longer acting molecules which are now in clinical development [13], and which may be the basis of the next generation of therapies, active against artemisinin-resistant malaria. The pattern is consistent: initial identification of natural product scaffolds, followed by their modification for clinical use by a combination of medicinal chemistry, formulation development and combination therapy.

The continual emergence or threat of emergence of drug resistance means that there will always be a need for new classes of molecules to combat malaria. The relative lack of success in finding new potent natural product scaffolds, could be taken to suggest natural products will be relatively less prominent in the future. However, before jumping to a premature conclusion it is important to focus on the other potential advantages of natural products.

(a) Natural products represent a source of potential new pharmacophores, the warheads that are needed for killing the parasite. Here it is important to distinguish between new pharmacophores and those which are already known to be cytotoxic. The latter are often seen as ‘frequent hitters’ in cellular screens (aptly named PAINs or pan-assay interfering compounds [14]) including tannins and polyphenols. It is also important to underline that many of the exotic chemical structures are chemically reactive Michael acceptors, which often are toxic due to irreversible covalent modification of host proteins and organs. What success really looks like is a novel molecule, which would give a new chemical insight, represent a novel mechanism of action, such as the artemisinin endoperoxide. It has to be underlined that many new scaffolds found in natural products will raise some concerns with medicinal chemists: quinine and artemisinin would be good examples here. These concerns are only going to be overcome by data, evidence of a wide cellular selectivity window, and therapeutic window in preclinical animal models.

(b) Herbal medicinal products are often perceived by the patient community as being safe, and many have been used by communities in disease endemic countries for generations. This safety question is not restricted to neglected diseases. In the US over 19% of the adult population use a herbal medicinal product[15], and the regulatory authorities have been obliged to develop a position on such products. Long-term medicinal or food use is currently accepted by some regulatory authorities as evidence of an adequate acute safety profile [16]. However, there are few studies of chronic safety data for most herbal medicinal products. This lack of well-documented safety data is concerning in the context of malaria, given that most patients are in vulnerable populations such as small infants and expectant mothers. Even though malaria is an acute disease, it is important to demonstrate safety after repeat dosing. In many parts of Africa, a child can have more than ten episodes of malaria per year. Significant caution must therefore be taken when assessing the chronic safety liabilities, especially questions around genotoxicty. This is illustrated by examples such as the herbal medicinal product aristolochic acid, incorrectly prescribed for weight loss (not an indication recommended by the traditional healers). Initial safety concerns were raised by acute renal failure in some patients [17]. The long-term perspective is equally distressing, as bladder cancer being observed in patients fifteen years after cessation of treatment [18]. This case serves as a reminder that plant secondary metabolites are generally produced as part of the host defence mechanism, and are likely to have some deterrent effect. The objective in anti-malarial therapy is to identify compounds which are selectively toxic against the parasite rather than the host.

(c) There is a strong desire on the part of the malaria endemic countries to be more active in using their ethnopharmacological heritage to identify new medicines to combat malaria. For example, the recently formed African Network for Drugs and Diagnostics Innovation (ANDi) has highlighted the role African countries can play in discovering new medicinal products [19]. In their 2009 meeting, half of the projects presented described the activities of un-purified natural products. Such projects fall outside the core activities of product development partnerships such as Medicines for Malaria Venture. However, support for such research activities by governments of disease endemic countries via organizations such as ANDi would be consistent with the pledges of the signatories of the 2000 Abuja declaration to increase support for research (including operational research) to develop new tools and improve existing ones. The development of herbal medicinal products are one of the areas where researchers from disease endemic countries can have a large impact [20]

(d) Herbal medicinal products are used by the community. This means that there is anecdotal evidence of their efficacy by the community. This is actually the key success factor. Its weight is strengthened if use of the same active ingredient has been documented to be used by several communities. If the definition of what is safe and active in human can be standardized, and the clinical activity defined, and plasma samples from patients can be shown to have *ex-vivo* activity, decisions can be made at an early stage which products to work on and purify further. This would allow a substantial focusing of natural product related drug discovery.

## Back to the roots – retracing how to find interesting natural products

**Figure 1:**
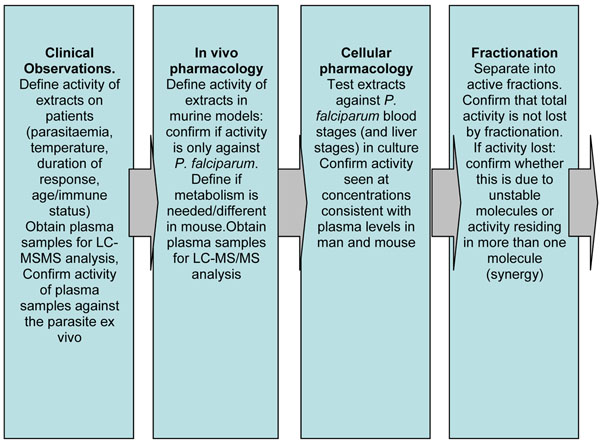
**A high level scheme for identifying natural products based on ethnopharmacology**. A high level scheme for identifying natural products based on ethnopharmacology. Screening strategies should be led by the initial confirmation that extracts have pharmacological activity at some level in the human disease and a clear absence of acute adverse events. Confirmation of such safety and efficacy from more than one study and community would be an advantage. Such a scheme would focus natural products research, prioritising those with confirmed activity in man. This process is an updated version of Yu Yunxiu’s 1952 doa-xing-ni-shi (acting in the reversed order) or 54321 [25]

The key question then becomes whether success with natural products can be improved by starting with extracts which have associated ethno-pharmacology data; those suggested to be active by the local traditional healers. The first hurdle to overcome is to confirm that the extract has some activity in patients (Figure 1). Many investigations fail at this point, taking for granted that their understanding of the clinical activity in some way relates to the understanding of the traditional healer. Before embarking on a costly and extensive characterization of a root or bark extract, it would be better to have confirmation that the extract is active in controlled studies in addition to the anecdotal reports.

**Figure 2:**
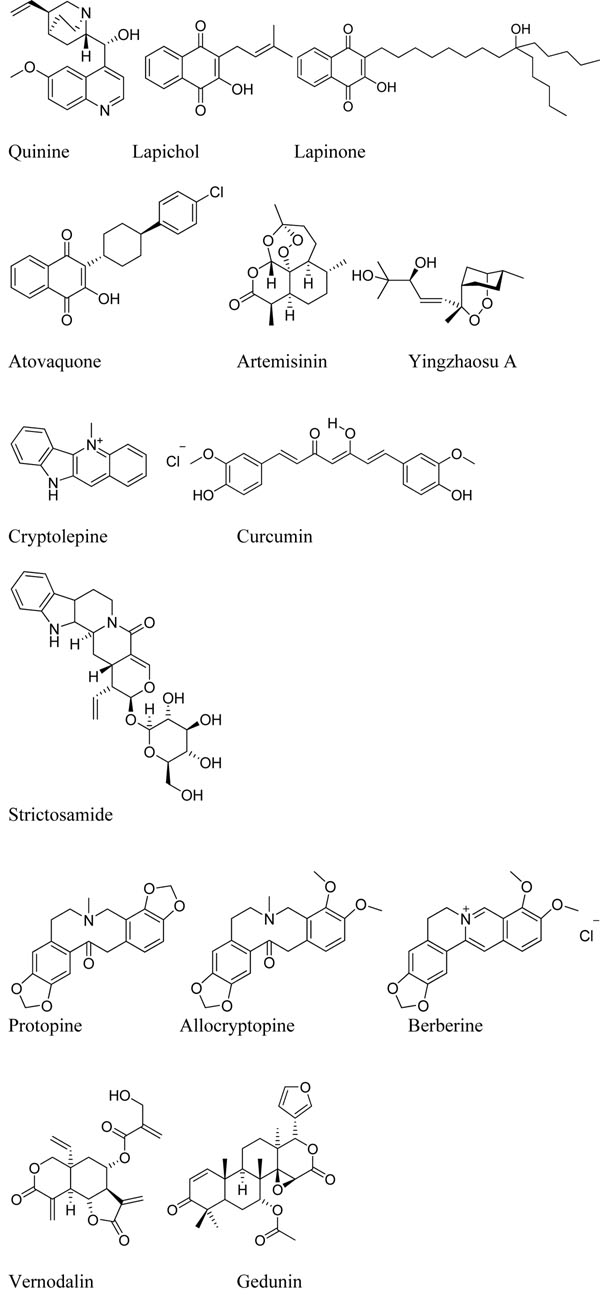
Structures of molecules from Herbal Medicinal Products with confirmed or presumed activities in human

Observation of pharmacological activity and safety in patients with malaria must be carried out within strict ethical guidelines [21]. Studies should be performed where the plant extract is the preferred treatment [22], there is a long history of use, well documented side effect profiles, and all subjects should give informed consent. Understanding safety is paramount, and so questions about side effects, contraindications, use in pregnancy and treatment duration need are essential [23]. Extracts which have been claimed as active by more than one community are more likely to yield a coherent molecular hypothesis. There needs to be a well documented method for pharmacognosy; the identification of the plants and the preparation of the extract, supported with an analytical profile of the extract. Close follow up and rescue therapy for treatment failures are essential. In order to separate anti-malarial activity from simple anti-pyretic activity, WHO diagnostic criteria for malaria infection of >2,000 parasites/ml in high transmission areas, (>1,000 parasites/ml in low transmission areas), temperature above 37.5 ^o^C and no concomitant infection should ideally be applied. In Africa, the semi-immune status of patients greater than five years old, will have a significant impact on cure rates at day 14, rendering rates of < 50% of questionable significance (see reference [24], Table 4). Therefore, it is important to know the age distribution of the patients (< 1 year old, between 1 and 5 years and over 5), the dose response and to know what proportion of patients achieved adequate clinical and parasitological response (ACPR) up to day 28. Although current WHO guidelines require treatments to have an APCR of >95% at day 28 [25] for new combination treatments, this is too rigorous for analysing the initial clinical data of natural product extracts. The expectations for the initial activity need to be more realistic than this, and based on historical successes with earlier generations of natural products. Certainly neither *Cinchona* nor *Artemisia* extracts on their own would meet these criteria. Purification, chemical modification, formulation and combination of these natural products produces improved medicines with >95% cure rates after three days of treatment, but this has taken many years of development work. The same process of optimization will be required for any new therapeutically active natural products. Finally, the identification of the active metabolite is very important, and it would be good to retain plasma and urine samples for metabolite analysis. Table 1 shows details of some of the natural products, which have been confirmed in observational clinical studies. As can be seen, often several of the key clinical parameters have been obtained, but rarely are all the key parameters for an observational study available. There is a clear need to define guideline protocols for such studies, as well as a central database of ongoing studies. The molecular details of the main active ingredients are also given. Here there is less clarity, since there are limited data about the activity of purified compounds *in vivo*, and there is plenty of scope in many of these molecules for interesting metabolic transformations carried out by either the gastrointestinal tract or hepatic metabolism in the human patient. This point is critical: the active ingredient may not be present in the initial herbal medicinal product, and its production may be restricted to processes which only occur in the human absorption or human metabolism. A standard analytical process for plasma, urine and plant samples would also be a great asset here along with standardized testing of the purified molecules. In addition a standardized process for testing plasma samples from patients in these studies against parasites *ex vivo* would be highly beneficial.

**Table 1:** Natural Products and extracts with confirmed or presumed activities in human

Natural product	Source	Mechanism of action	Highest level demonstration of activity
Quinine	*Cinchona* genus	Assumed to be similar to chloroquine – and prevent heme polymerisation.	Early reports of activity with *Cinchona* bark showed partial activity with 60g bark over up to 21 days. This represents a total dose of 350 -700 mg, compared to a current clinical dose of 500 mg (10 mg/kg salt) given three times a day for seven days currently - suggesting the bark treatment was unlikely to be completely effective [31]

Lapachol Lapinone Atovaquone	*Bignoniaceae*	Electron transport inhibition	Lapachol is a naphthoquinone used to treat malaria and fevers [41] reported in the 19^th^ century; it showed weak activity against *P. zophurae* infected ducks, when tested in 1943. Lapinone (a close derivative) was confirmed active in patients with *P vivax*[42]by intravenous administration for four days (N=9). Chemical optimisation of the scaffold led to atovaquone

Artemisinin	*Artemisia annua*	Free radical activation in the presence of free ferrous iron – liberated in erythrocytes by parasite digestion of haemaglobin	Traditional Chinese Medicine. Tea made from 5 g/l leaves gave 12 mg artemisinin, and clears parasite in 3-4 days [43]. (Partial protection, since this is much lower than the WHO recommended dose). More recent studies [44] achieved doses of 95 mg, with 70% cure on day 7, but still with high recrudesence and only 30% cure at day 28 [45]. Chemical optimization has led to longer acting synthetic endoperoxides, currently in development.

Yingzhaosu A	*Artabotrys uncinatus* (Ying Zhao)	Presumed to be free radical activation in the presence of free ferrous iron – liberated in erythrocytes by parasite digestion of haemoglobin.	Traditional Chinese medicine. The active ingredient was modified to make Ro-41-3823 which was: Single tested in patients (N=30) aged 12 -42 years, with parasitaemia > 5000/ml and temperature 37.7 -39.8 ^o^C. 80% patients were parasite free at day 7 with a single dose of 25 mg/kg.[46] Discontinued because of lack of superiority over mefloquine or artemisinin, and because of safety concerns.

Cryptolepine	*Cryptolepis sanguinolenta*	DNA intercalation [47]	Patients between 16 and 60, (N=12) with parasitaemia between 1000 and 10000/ul given 25 mg/kg extract tid for seven days. No recrudescence at day 28. Cryptolepine administered orally to *P.* berghei-infected mice in doses of 50mg/kg/day for four days reduced parasitaemia by 80% but the mice were not cured of malaria (Wright et al., 1996) [48]. Decoction has been standardized by the Faculty of Pharmacy, Kwame Nkrumah University of Science and Technology, Ghana and is marketed as PHYTO-LARIA®.

Curcumin	*Curcuma longa*	Antioxidant activity?	45 patients have been treated with a nanomilled curcumin, both vivax and falciparum malaria. Nanomilling is used to improves bioavailability. No clinical data on parasitaemia or fever available S Kumesh Kar *pers. comm*

Strictosamide	*Nauclea pobeguinii*		Traditional treatment from DR Congo, Herbal medicinal Product PR 259 CT1 – completed a phase I trial – 1000 mg t.i.d for 7 days [49] Phase IIb (N=65 patients) treated with 1000 mg tid for three days, followed by 500 mg tid per day for four days. Parasitaemia fulfilled the WHO research criteria for malaria. ACPR at day 14 was 90.3% compared with ASAQ at 96.9%*.*[*50*]. Shown to be orally active in murine models but not *in vitro* suggesting that deglycosylation may be required for activity.

Protopine Allocryptopine Berberine	*Argemone mexicana*		Initial study (N=80) with 80% patients < 5 years old. Showed need for high dose regimen [51]. Follow up study (N=199) vs amodiaquine artesunate (N=102) [52] median age 5 years given for 7-14 days twice per day 89% successful at day 28, compared with a 95% success rate for ASAQ. P atients had low parasitaemia (<1000/μL), with only 17.8% of patients fulfilled the WHO research criteria for clinical malaria.

Vernodalin	*Vernonia amygdalina*		Known as omubirizi in southwestern Uganda and used for pain relief and malaria attack, obtained from The Medical Traditional Healer Association in Rukararwe, Bushenyi District, Uganda [53] Clinical study for decoction (N=33) infusion given four times per day for 7 days [54] 67% response, inclusion criteria allowed patients with <2000/uL and temperature <37.5 ^o^C. All patients over 12 years old

Febrifugine	*Dichroa febrifuga*		*Ch’ang shan*, is a traditional Chinese anti-malarial herb. Reports from 1942 suggest a dose of 60 mg was clinically effective [55]. Adverse reaction prevented widespread use of febrifugine. Halofuginone, a halogenated derivative is used against coccidiosis, and other derivatives have been tested against Plasmodium [56]

Gedunin	*Azadirachta indica*	HSP90 inhibitor ?[57]	Neem extracts are known to be active based on traditional and observations from India in the early 20^th^ century [58]. No published recent clinical studies on malaria, although the aqueous/acetone extract is safe [59] and is registered in Nigeria in 250mg capsules as IRACARP®, adult treatment costs of around $6.00. Most potent ingredient is gedunin [60]. Murine activity is variable and requires cytochrome 3A4 inhibitor.[61]

There are two well documented commercial uses of herbal medicinal products. The first by the Department of Medicaments traditionnels améliorés, (DMT) in Mali which uses a mixture of three herbs *Cassia occidentalis* (leaves), *Lippia chevalieri Moldenke* (leaves), and *Spilanthes oleracea*, *Jacq* (flowers). Sidibe OM, (2006) Pharmacy Thesis, University of Bamako, Mali *http://www.keneya.net/fmpos/theses/2006/pharma/pdf/06P18.pdf*.

Ayush-64 is a combination of four Ayurvedic drugs used in India registered by the Central Council for Research in Ayurveda and Siddha (CCRAS). It is formulated into tablets, and dosed at 1g per day over 5-7 days, at a treatment cost of 14 rupees ($0.28) [62]. Effectiveness, measured as ACPR at day 28 was only 48.9% compared with 100% for chloroquine, and it is not recommended for use in treatment. However, the activity in man suggests that there is a need for identification and optimisation of the active ingredients or their metabolites. This figure builds from Table 3 from Willcox and Bodeker 2004 [63]

## *Dao-xing-ni-shi*

The approach of starting with the activity in humans was suggested in 1952 by Chen Guofu when working on the Chinese herbal medicinal product *Ch’ang shan* and was labelled *dao-xing-ni-shi* or ‘acting in the reversed order’ [26]. At the time there were major objections by some in the clinical community who saw this as merely using patients as guinea pigs, and there were additional concerns regarding using un-purified materials. In the intervening sixty years, several factors have ameliorated these concerns. First, acceptance by authorities such as the European Medicines Agency, the US-FDA and the WHO, that since patients and communities use herbal medicinal products, there is a public health responsibility to understand them better and produce guidelines around their preparation, usage and safety. Some disease endemic country regulatory authorities have already approved herbal medicinal products, and so carry an addition responsibility to ensure ongoing data collection on clinical safety and efficacy. Second, the assays for testing the anti-malarial activities of samples are now extremely robust, and can be run with relatively low volumes. This means that any plasma or urine sample taken from a patient can be initially tested to see if it has anti-malarial activity *ex-vivo*. Third, analytical methodologies and technologies are available which allow detailed analysis of the plant material by liquid chromatography and mass spectrometry, and a comparison with plasma and urine samples from patients. Combining these approaches with our growing understanding of xenobiotic metabolomics, offers the possibility to identify the active ingredients in human. Recent advances in analysis coupled with statistical analysis and mathematical modelling provide a baseline for such studies [27,28]. It may well be that the original herbal medicinal product has to be metabolized in the gastrointestinal tract or liver to produce the active ingredient. In this case, the original extract would not have a high degree of activity in cell-based assays. It may also have sub-optimal activity in murine *in vivo* assays, if either there are significant differences in either the murine metabolism or potency against rodent parasite. There are many examples in malaria therapy of metabolism being required for activity. 8-aminoquinolines are active against the blood and hepatic stages of *Plasmodium* but require hepatic metabolism [29]. Extracts from *Nauclea pobeguinii* are active in both rodent models and patients, despite none of the alkaloids showing *in vitro* activity, suggesting activation of strictosamide, probably by the gastrointestinal tract, is necessary for activity [30].

There is a strong interest from scientists in disease endemic countries to work on their traditional medicines. With the emergence of organizations such as ANDi, there is the possibility of investments in the infrastructure and methodology to enable best practice for these observational studies to be shared across disease endemic countries, as well as the analytical and data interpretation platforms, which cannot be provided by every disease endemic country.

## Potency guided identification of active ingredients

Once a significant clinical response has been verified, the active entities must be chemically identified and characterized. Of course, the natural product extract could be used directly as a decoction, and in many countries such extracts are sold commercially. However, the history of malaria chemotherapy with quinine, lapinone and artemisinin shows that more potent and long acting medicines can be discovered if the active ingredient is identified and used as the basis of a medicinal chemistry program. Purification also allows the separation of the active ingredient from non-active, but toxic molecules. It should not be forgotten that after self-administering cinchona bark extract, in the nineteenth century Christian Hahnemann invented the concept of homeopathy (infinitely dilute solutions of toxins being proposed as a cure). The *cinchona* extract that was supposed to treat malaria gave him malaria-like symptoms of palpitations, drowsiness, vomiting and feeling cold (caused by quinidine and other alkaloids), leading him to coin the concept *similia similibus curentur* ‘like cures like’ [31].

Activity based purification requires an assay, and it is important have an assay which is robust, medium throughput, and reflects the clinical situation. If the purification starts with extracts known to be clinically active in man, the process is much easier. Before moving to *in vitro* assays it is important to confirm the clinically observed activities *in vivo* (Figure 1), to understand better metabolic issues*.* The standard *in vivo* models of infection include the infection of mice with *P. berghei*[*32*] and *P. yoelii*. Of course, if the mouse model does not confirm the human clinical and anti-parasitic activity, then the issue is with the murine models, and could be due to three factors. First, the parasite species is different; and the compound is less potent against the murine parasite than against *P. falciparum.* This difference in potency has been seen in the analysis of pharmaceutical ‘hits’, and can be discriminated using humanized or SCID mouse models, which can be infected with *P. falciparum*[*33*]. This model is technically more challenging, and currently four times as expensive, but could be usefully deployed where the human clinical data around an extract are reasonably solid. Second, as outlined above, host metabolism may differ in terms of the extent and nature of any metabolism; murine models with human hepatocytes are under development [34], and this may be of help in the future. For the moment, the best data would be obtained by comparing LC-MS/MS data and bioactivity between human and murine plasma samples, and also by examining the impact of liver microsomes on the molecules in the decoctions. Third, the pharmacokinetics may be different in the two systems; most molecules have a much shorter half-life in mouse than in human, and this may require even more frequent administration than was used clinically, and usually means higher doses. Once activity is confirmed in murine models, then either the complete extract can be tested on cellular models or the extract can be fractionated for testing in cell-based assays *in vitro*. Caution is needed here. If it is known that the patient plasma samples have *ex vivo* anti-parasitic activity, and if the final active ingredient does not have activity at a concentration which is achieved in plasma during the clinical study, then it is unlikely to be the real active, unless pharmacological synergy is occurring. The hundreds of projects where molecules have been purified and shown to be active in the only in the10 – 100 uM range fall into this category. Significant resources are being used to identify molecules which are unlikely to be useful. By resetting the thresholds to focus on more potent molecules, and insisting on observational clinical data and *ex vivo* analysis, the entire process could be streamlined, and this pitfall largely avoided.

Once the active ingredient is identified, then it will still need some optimization. Moving from quinine via methylene blue to chloroquine gave significant improvement in frequency of administration; moving from artemisinin to artesunate allowed a decrease in dose, and more consistent bioavailability. This optimization work will most probably be needed with all new herbal based actives anti-malarials: the perception that ‘Nature’ has designed the magic bullet, which just needs to be identified, produced and marketed, is far from the historical experience.

It is often suggested that in herbal medicinal products, more than one active ingredient may be required for pharmacological activity. This is often given as a reason for the lack of progress in the identification of new natural product templates for anti-malarial agents. Although this is attractive to many in the herbal medicinal products world, the data to support synergy of more than one sub-therapeutic ingredient is extremely sparse in the literature. The three cinchona bark alkaloids quinine quinidine and cinchonine were shown to have mild synergy against some, but not all strains tested *in vitro*[*35*]*.* However, this synergy is not significant clinically [36]. The synergy when seen was not sufficiently large that activity was lost on fractionation (a typical argument why the reductionist approach does not work. In reality, if biological activity is lost on fractionation it results from inactivation of the active molecule (oxidation, chemical break down). Demonstration that two active ingredients are absolutely required for activity means that two of the inactive fractions have to be recombined to give the activity. In addition, it is important to differentiate synergy from the presence of solubilizing or stabilizing factors. Artemisinin is a good case in point; its potency and solubility can be improved by the chemical modification to artesunate. In the plant, the solubility limitation is overcome by solubility potentiation by the flavone casticin [37], or protection by anti-oxidants [38]. This is more a case of formulation development than pharmacological synergy. Strictly speaking synergy requires two active ingredients, that when combined have a resultant activity greater than the sum of the two ingredients on their own – casticin’s anti-malarial activity is only 24 mM. Clinical synergy could also take place as at the level of exposure levels, one natural product inhibiting the metabolism of another in the mixture.

It has been often suggested that crude extracts of *qinghaosu* are natural a ‘artemisinin combination therapy’, (playing on the perceptions amongst patients that ACT is an excellent medicine, but somehow ‘natural’ medicines are better), but this remains to be demonstrated. The goal of combination therapy from the viewpoint of the WHO is that one drug should protect the other against resistance. Both ingredients, therefore, need to have intrinsic anti-parasitic activities and different modes of action and mechanisms of resistance. Unless a natural product extract has two or more such components, it is acting as a monotherapy. A strategy to disseminate such monotherapy decoctions raises the concern that it may facilitate the generation of resistance, combining under-dosing with monotherapy [39], however this danger needs to be balanced with the observation that the Chinese used this decoction for centuries without generating resistance.

Of the thousands of herbal medicinal products which have been suggested to be active for malaria, only a very few have been clearly demonstrated to be active in patients and animal models prior to fractionation. These are summarized in Table 1. One reason for this dearth is simply a lack of funding; but it is a lack of focus. Modern synthetic organic chemistry grew from natural products; most organic chemistry projects in the twentieth century involved identifying and structurally characterizing a natural product, and then confirming this structure by synthesis. Confirmation of biological activity often comes as an afterthought, rather than a prerequisite. The available of mass spectrometry technologies allowing more rapid characterization of natural products has further dissociated structure from function. There is often a disconnection between natural products chemistry and clinical and pharmacological reality in practice, a gap which needs to be addressed.

## Marine, fungal and bacterial natural products

Going back to the ethnopharmacological roots is not an option for two categories of natural products: those from marine organisms, and those from fungal fermentations. Work on marine organisms is still in its infancy compared to plant natural products, with the first medicine being launched over the last seven years (cancer and chronic pain) [40]. Microbial fermentation products continue to be of interest, several groups have worked on trying to identify a macrolide with better anti-malarial activity than azithromycin. Screening the diversity collections of Japanese pharmaceutical companies may throw more light on this question, since they have been slower to abandon microbial natural products than their US or European counterparts. However, it is still important to retain the same standards for submicromolar potency and truly novel chemistry.

## Networks for success

Partnerships such as Medicines for Malaria Venture (MMV) and Drugs for Neglected Diseases Initiative (DNDi) have proved their ability to develop and deliver new anti-malarial medicines: products from their partnerships treated over fifty million children in the last 12 months. However, the leadership in the identification and characterization of herbal medicinal products requires local knowledge and sensitivity. It implies deep involvement of the Health Ministries and Drug Registration Authorities of the disease endemic countries, since many countries already approve the sale of such products. Sovereignty issues around natural products also argue strongly that these initial endeavours are best supported locally, in countries investing in their own patrimony of ethnopharmacology, perhaps supported with technology co-ordination and financial support from organisations such as the African Network for Drugs and Diagnostics Innovation (ANDi). Some malaria endemic countries will consider funding such ethnopharmacology-based programmes as a way of fulfilling their Abuja commitments to malaria research and development. For now, the key gap is to fund collaborations with a bigger picture; combining natural products and observational clinical studies from the start. Longer term it means bringing together the networks of plant scientists, clinicians, pharmacologists and analytical chemists to form productive collaborations, and deliver new medicines.

## Conclusions

All of the medicines that are used today against malaria come from natural product lineages which can be traced back to herbal medicinal products: quinine, lapachol and artemisinin. From each of these starting points, new innovative molecules have been developed by medicinal chemistry, formulation development and combination therapy that are better suited to the needs of the patients. The trend over the last few decades has moved towards a reductionist ‘smash and grab’ approach to natural products, which has not delivered the same success. This recent success of cell based screening over target screening for pharmaceutical libraries is forcing us to re-evaluate our approach to drug discovery. Herbal medicinal products carry one strategic advantage – that in several cases we know that there is an activity in patients. Once the clinical activity of a herbal medicinal product is verified in observational studies, the anti-parasitic activity of the plasma samples on parasites could be confirmed *ex vivo*, and characterization of the decoction and the plasma samples using mass spectrometry and HLPC separations. Once the active ingredients are identified it is likely that medicinal chemistry will be needed to optimize it for clinical use. Disease endemic countries play a key role in this process. Countries with registered Herbal Medicinal Products have a responsibility to their patients to understand the mode of action better. Organisations such as the African Network for Drugs and Diagnostics Innovation (ANDi) can play a key role in helping to standardize the clinical protocols used to understand herbal medicinal products, as well as providing the technology network to assist the activity guided purification of the active ingredients. Natural products have been the starting points for new malaria medicines over the last century. By making the most of the potential for clinical data, it is possible that they could continue to influence our thinking for the next century.

## List of abbreviations used

Artemisinin combination therapy (ACT); African Network for Drugs and Diagnostics Innovation (ANDi)

## Competing interests

The author declares that he has no competing interests.

## Authors' contributions

This manuscript was conceived and written by TW.

## Acknowledgements

The author thanks MMV and its advisory committees for their comments in the preparation of this document, especially Jeremy Burrows, Simon Croft, Brian Greenwood, Winston Gutteridge, François Nosten, Carol, Sibley and also to Sir Colin Dollery and Matthias K. Schwarz. Special thanks to the Traditional Medicinal Plant community, especially Merlin Willcox and Bertrand Graz for their helpful comments and criticism, and to Solomon Nwaka for his leadership and inspiration of ANDi. The views expressed here are those of the author, to serve as the basis for discussion, and should not be interpreted as the approved strategy Medicines for Malaria Venture.
